# The role of sphingolipid rheostat in the adult-type diffuse glioma pathogenesis

**DOI:** 10.3389/fcell.2024.1466141

**Published:** 2024-12-11

**Authors:** Ivana Karmelić, Mia Jurilj Sajko, Tomislav Sajko, Krešimir Rotim, Dragana Fabris

**Affiliations:** ^1^ Department of Medical Chemistry, Biochemistry and Clinical Chemistry, School of Medicine, University of Zagreb, Zagreb, Croatia; ^2^ Department of Neurosurgery, University Hospital Center “Sestre milosrdnice”, Zagreb, Croatia

**Keywords:** diffuse glioma, glioblastoma, sphingosine-1-phosphate, ceramides, sphingolipid rheostat

## Abstract

Gliomas are highly aggressive primary brain tumors, with glioblastoma multiforme being the most severe and the most common one. Aberrations in sphingolipid metabolism are a hallmark of glioma cells. The sphingolipid rheostat represents the balance between the pro-apoptotic ceramide and pro-survival sphingosine-1-phosphate (S1P), and in gliomas it is shifted toward cell survival and proliferation, promoting gliomas’ aggressiveness, cellular migration, metastasis, and invasiveness. The sphingolipid rheostat can be altered by targeting enzymes that directly or indirectly affect the ratio of ceramide to S1P, leading to increased ceramide or decreased S1P levels. Targeting the sphingolipid rheostat offers a potential therapeutic pathway for glioma treatment which can be considered through reducing S1P levels or modulating S1P receptors to reduce cell proliferation, as well as through increasing ceramide levels to induce apoptosis in glioma cells. Although the practical translation into clinical therapy is still missing, sphingolipid rheostat targeting in gliomas has been of great research interest in recent years with several interesting achievements in the glioma therapy approach, offering hope for patients suffering from these vicious malignancies.

## 1 Introduction

Gliomas, the most prevalent and aggressive form of intracranial cancer, account for over 80% of malignant brain tumors, with glioblastoma multiforme (GBM) as the most common and severe subtype (Ostrom et al., 2014). Despite the significant research interest and certain progress in glioma treatment in recent years, the prognosis for patients with gliomas, especially GBM, remains very poor (Ma et al., 2024). The latest fifth edition of the World Health Organization (WHO) tumor classification of the central nervous system (CNS) introduced significant updates, incorporating molecular and genetic parameters for more precise clinical categorization (Louis et al., 2021). Sphingolipids, especially glycosphingolipids, are abundantly in the brain and integral to cell membrane structure and cellular signaling (Hannun and Obeid, 2018). Abnormalities in sphingolipid metabolism have been implicated in promoting gliomas’ aggressiveness, strongly affecting numerous characteristics of tumor phenotype, such as cellular migration, metastasis, and invasiveness (Zaibaq et al., 2022). Their main metabolic enzymes have been actively investigated as new potential targets in glioma therapy development (Wang et al., 2022). The “sphingolipid rheostat” concept highlights the opposing roles of ceramide, which induces apoptosis, and sphingosine-1-phosphate (S1P), which promotes proliferation (Cuvillier et al., 1996). This balance between the pro-apoptotic ceramide and pro-survival S1P is crucial in determining the cell’s fate, and in glioma it is altered towards the pro-survival S1P signaling, promoting uncontrolled cell proliferation and invasiveness (Zaibaq et al., 2022). This mini-review explores the complex and interconnected metabolic effects of sphingolipids in glioma progression, focusing on new achievements in sphingolipid rheostat targeting as an innovative glioma therapy approach, alone or combined with the established current glioma therapies to improve glioma, particularly GBM patient outcomes.

## 2 Adult-type diffuse gliomas classification

**FIGURE 1:**
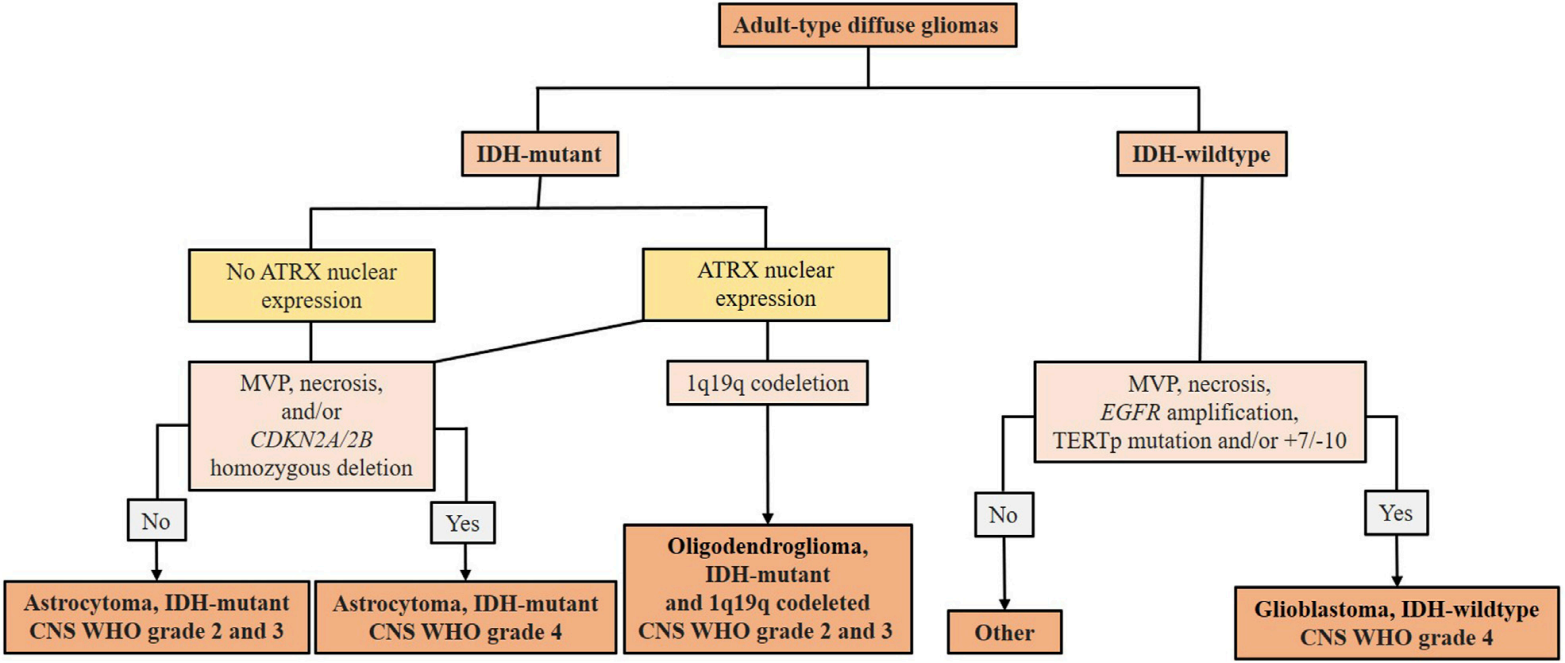
Summary scheme of the 2021 WHO classification of adult-type diffuse gliomas (according to Martin et al. (2023)). After the identification of adult-type diffuse glioma, the presence of IDH mutation must be evaluated. In IDH-mutant gliomas, nuclear expression of ATRX is immunohistochemically evaluated. In the absence of ATRX expression, it is classified as astrocytoma, IDH-mutated WHO CNS grade 2 or 3. Astrocytoma, IDH-mutated WHO CNS grade 4 is defined by the presence of MVP, necrosis, and/or *CDKN2A/2B*. If nuclear ATRX expression is conserved and 1p19q codeleted, it is characterized as an oligodendroglioma, IDH-mutated and 1p19q codeleted. Without 1p19q codeletion, it remains astrocytoma, IDH-mutated. IDH-wildtype gliomas are classified as GBMs in the presence of MVP, necrosis, *EGFR* amplification, *TERT* promoter mutation, and/or +7/−10. IDH-wildtype gliomas that do not meet these diagnostic criteria should be investigated for other potential types of glioma tumors. IDH (isocitrate dehydrogenase) - mutations cause the overproduction and accumulation of the 2-hydroxyglutarate, an oncometabolite that promotes DNA methylation leading to changes in the epigenetic status and blocking cellular differentiation (Zaibaq et al., 2022); *ATRX* (α thalassemia/mental retardation syndrome, X-linked gene located in Xq21.1) - loss of function is associated with telomere elongation leading to stronger cellular proliferation (Byun and Park, 2022); MVP (microvascular proliferation); *CDKN2A/2B* - tumor suppressor genes located on chromosome 9p21 involved in cell cycle regulation; their homozygous deletion indicates a higher tumor grade (Louis et al., 2021); 1p19q codeleted - concurrent loss of the short arm of chromosome 1 (1p) and the long arm of chromosome 19 (19q); *EGFR* (epidermal growth factor receptor gene) amplification can lead to overexpression of EGFR protein associated with aggressive behavior of gliomas (Bassi et al., 2021); *TERT* (telomerase reverse transcriptase) promoter mutation can lead to increased activity of telomerase and are associated with enhanced cellular proliferation and tumor progression (Byun and Park, 2022); (+7/−10) - combined whole chromosome 7 gain and whole chromosome 10 loss.

Diffuse gliomas are the most common type of primary CNS tumors in adults (Lucke-Wold et al., 2024; Martin et al., 2023). The fifth edition of the WHO classification of CNS tumors (CNS5) introduced significant changes by adding molecular and genetic markers to the previously histology based classification, making it more precise and clinically relevant (Louis et al., 2021). According to WHO CNS5, there are three main types of adult-type diffuse gliomas: (I) astrocytoma, isocitrate dehydrogenase (IDH)-mutant; (II) oligodendroglioma, IDH-mutant and 1p/19q codeleted; and (III) glioblastoma, IDH-wildtype (Louis et al., 2021; Gisina et al., 2022) (Figure 1). IDH-mutant diffuse astrocytic tumors are considered as astrocytoma, IDH-mutant CNS WHO grades 2, 3, or 4 (Louis et al., 2021). Oligodendrogliomas, IDH-mutant and 1p/19q codeleted are classified as CNS WHO grade 2 or 3 (Louis et al., 2021). IDH-wildtype diffuse astrocytic tumors are now classified as GBM CNS WHO grade 4 if there is microvascular proliferation (MVP) or necrosis and if they meet 1 or more of 3 specific key molecular criteria (Figure 1), even if they show low-grade histological features (Louis et al., 2021). Further evaluation is necessary to classify other IDH-wildtype gliomas that do not meet these molecular criteria (Louis et al., 2021; Meyer et al., 2021). Astrocytoma, IDH-mutant CNS WHO grade 4 are no longer classified as GBMs due to distinct molecular and epigenetic profiles (Figure 1) and different clinical behaviors (Zaibaq et al., 2022; Rajaratnam et al., 2020). The most malignant and aggressive of the adult-type diffuse gliomas are the astrocytoma, IDH-mutant CNS WHO grade 3 and 4, and GBM, IDH-wildtype CNS WHO grade 4, considered higher-grade gliomas (HGG) (Martin et al., 2023). Glioblastoma accounts for nearly 15% of all brain tumors, with approximately 80% being primary tumors, mainly occurring in older patients, while secondary GBMs develop from lower-grade gliomas primarily in younger patients (Louis et al., 2021). Despite aggressive standard therapy, including radical surgery, radiotherapy and temozolomide (TMZ) chemotherapy, and even with adjuvant chemotherapy, the median overall survival of remains 14–20 months (Ma et al., 2024; Stupp et al., 2005). Other therapies, such as carmustine wafers placed in surgical cavity, anti-VEGF antibodies, PDGF and EGFR inhibitors, and immunotherapy, extend survival for only a few months (Norden and Wen, 2006). Recurrence remains nearly inevitable due to GBM’s aggressiveness and therapy resistance, driven by brain tumor-initiating cells (BTICs) (Landis et al., 2018). Lower-grade gliomas, such as astrocytoma, IDH-mutant CNS WHO grade 2 and oligodendroglioma, IDH-mutant and 1q19q codeleted CNS WHO grade 2, have a better prognosis, but their treatment is also challenging as most of them tend to progress to HGG over time (Yu et al., 2020).

## 3 Sphingolipid metabolism

Sphingolipids are ubiquitous components of eukaryotic cell membranes, especially abundant in human neural tissue, where they contribute (Gault et al., 2010) to membrane structure and serve as signaling molecules in processes like apoptosis, proliferation, angiogenesis, and vesicular trafficking (Ogretmen, 2017). They are commonly divided into simple, sphingoid bases and ceramides, and complex sphingolipids, such as glycosphingolipids, sphingomyelins and sulphatides (Hannun and Obeid, 2018).

Sphingoid bases, long-chain aliphatic amino alcohols, form the structural backbone of sphingolipids and serve as signaling molecules. Their chain length can vary from 12 to 26 carbons, with sphingosine (d18:1) and sphinganine (d18:0) predominating in mammalian tissue; sphingosine arise from sphingolipid degradation, while sphinganine is a precursor in *de novo* sphingolipid biosynthesis (Pruett et al., 2008).

Ceramides, bioactive sphingolipids are synthesized by six ceramide synthase isoforms (CERS1-6) which bind fatty acids of varying chain lengths to a sphingoid base, resulting in ceramides with diverse functions and tissue distributions (Riebeling et al., 2003; Raichur, 2020). In human brain tissue, 18-carbon fatty acid ceramides are the most abundant (Pruett et al., 2008). Ceramides are synthesized either *de novo* or via hydrolysis from complex sphingolipids and are further catabolized by ceramidases to form sphingoid bases, serving as a key intermediates and precursors for all complex sphingolipids (Zheng et al., 2006; Mullen et al., 2012). Bioactive sphingoid bases and ceramides disrupt pro-survival cellular signaling pathways inducing apoptosis by various activating mechanisms (Nganga et al., 2018). Extracellularly oriented ceramides produced by acid sphingomyelinase (sphingomyelin phosphodiesterase 1, SMPD1) in lipid rafts activate membrane receptor clustering with different tumor necrosis factors triggering strong apoptotic signal responses (Dumitru et al., 2009). Intracellularly, ceramides trigger apoptosis by affecting outer mitochondrial membrane permeability and regulating apoptosis-related molecules like phosphatases, kinases, and phospholipase (Gault et al., 2010; Dadsena et al., 2019) During oncogenic transformation, cells often evade apoptosis as ceramidases become more active, hydrolyzing ceramides into sphingosine and fatty acid (Levery, 2005).

**FIGURE 2:**
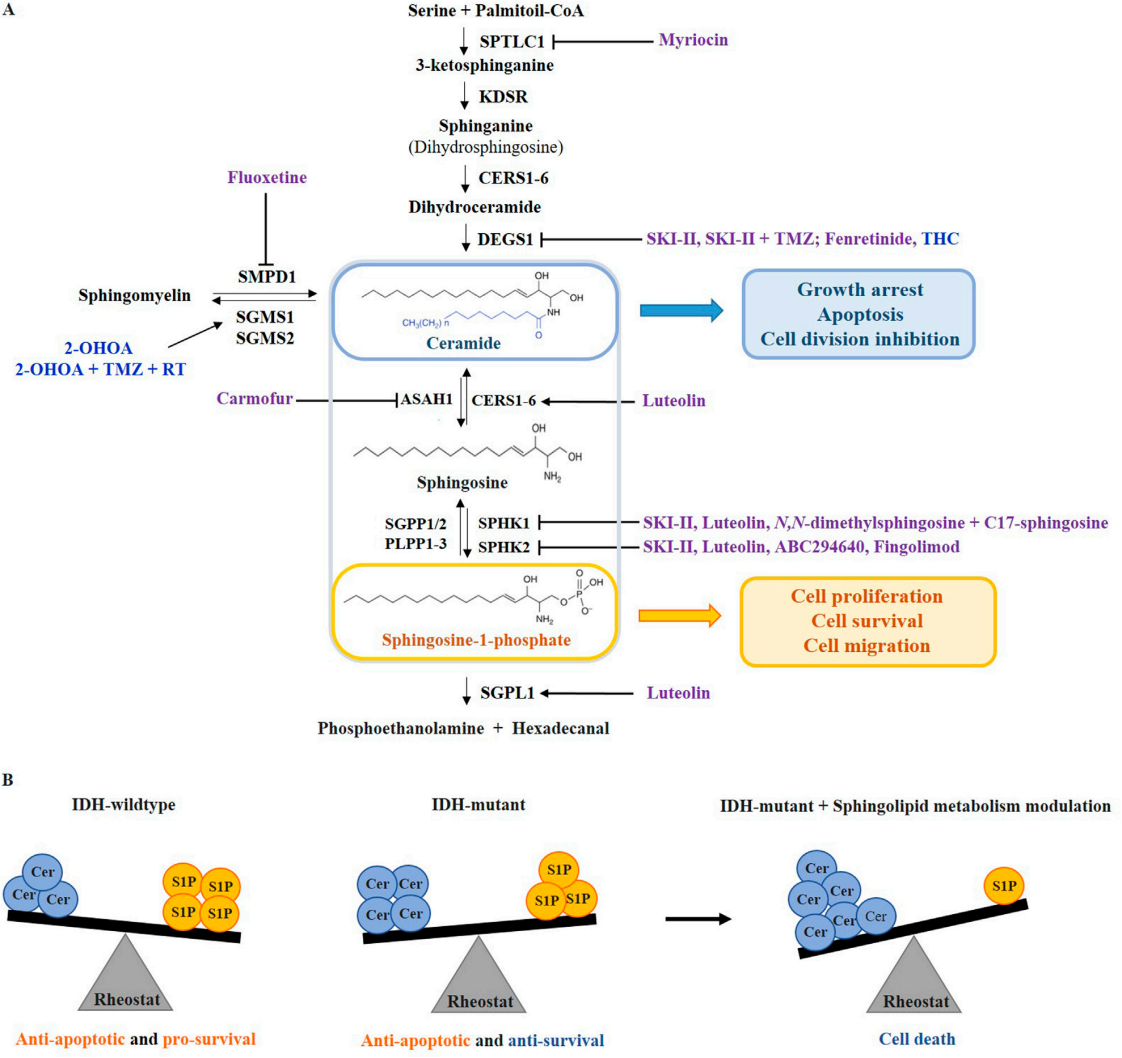
The sphingolipid rheostat targeting in adult-type diffuse gliomas. **(A)** The balance between sphingolipids that act as tumor suppressors (ceramide) and those that act as tumor promoters (sphingosine-1-phosphate, S1P) is controlled by targeting the enzymes of sphingolipid metabolic pathway. Drugs that have been involved in clinical trials on glioma patients are colored blue, while pre-clinical drugs are colored purple. **(B)** In IDH-wildtype gliomas, the sphingolipid rheostat is shifted towards high S1P levels, promoting anti-apoptotic and pro-survival signaling. In IDH-mutant gliomas, the sphingolipid rheostat is reversed but with preserved anti-apoptotic signaling. Due to the higher vulnerability of IDH-mutated gliomas, the drugs that induce ceramide accumulation or reduce S1P, can further alter the rheostat inducing cell death. SPTLC1 - serine palmitoyltransferase 1; KDSR - 3-ketosphinganine reductase; CERS1-6 - ceramide synthase 1–6; DEGS1 - dihydroceramide desaturase 1; ASAH1 - acid ceramidase; SGPP1/2 - sphingosine-1-phosphate phosphatases 1 and 2; PLPP1-3 - phospholipid phosphatases 1–3; SPHK1/2 - sphingosine kinase 1 and 2; SGPL1 - sphingosine-1-phosphate lyase 1; SMPD1 - acid sphingomyelinase 1; SGMS1/2 - sphingomyelin synthase 1 and 2; THC - ∆^9^-tetrahydrocannabinol; 2-OHOA - 2-hydroxyoleic acid; TMZ - temozolomide; RT - radiotherapy.

Sphingosine kinases 1 and 2 (SPHK1/2) phosphorylate sphingosine to S1P, with SPHK1 being localized in the cytoplasm and SPHK2 in the nucleus, endoplasmic reticulum (ER), mitochondria, and, in cancer cells, in the plasma membrane (Diaz Escarcega et al., 2021). While SPHK1 has mainly pro-survival roles, SPHK2 can promote both pro-apoptotic and pro-survival signals depending on its localization in the cell. SPHK2 can be translocated to the nucleus where the formation of S1P inhibits histone deacetylase 1 and 2 (HDAC1/2) activity, leading to epigenetic changes in gene expression (Diaz Escarcega et al., 2021). Sphingosine-1-phosphate act as a second messenger regulating cell growth, differentiation, migration, and apoptosis by binding to G protein-coupled receptors, S1PR1-5, with variable tissue expression and important roles in development, aging, and pathologies (Merrill, 2011; Sousa et al., 2023; Blaho and Hla, 2014). S1P controls various signaling pathways, including angiogenesis and proliferation, by activating cytosolic effectors, phospholipases, and kinases (Pyne and Pyne, 2000). It is inactivated by ER-enzymes S1P-phosphatases 1 and 2 (SGPP1/2) and phospholipid phosphatases 1–3 (PLPP1-3), or by S1P lyase 1 (SGPL1) localized in the ER and Golgi, but also in plasma membrane, where it directly influences extracellular S1P concentrations (Sousa et al., 2023; Tang and Brindley, 2020) (Figure 2A).

## 4 Sphingolipid rheostat in gliomas

The sphingolipid rheostat concept refers to the balance between pro-apoptotic ceramides and sphingosine, and pro-survival S1P, where ceramide and sphingosine are directly correlated to the downregulation of S1P and vice versa (Cuvillier et al., 1996) (Figure 2A). Bioactive sphingolipid metabolites may serve as biomarkers for cancer malignancy, progression, and metastasis (Newton et al., 2015) (Figure 2B).

Overexpression of SPHK1 and the resulting production of S1P correlated with malignancy, poor prognosis, and shorter survival time in different types of gliomas, especially GBMs (Young and Van Brocklyn, 2007; Li et al., 2022). Increased SPHK1 and decreased SGPP1/2 expression were a hallmark of cultured GBM cells with upregulation of SPHK1 promoting growth and metastasis of GBM cells (Zaibaq et al., 2022; Abuhusain et al., 2013). Upregulated acid ceramidase (*N*-acyl sphingosine amidohydrolase 1, ASAH1) and SPHK1 were detected in GBM tissues compared to normal brain as well as higher ASAH1 activity in BTICs (Abuhusain et al., 2013). S1P was reported to be, on average, 9-fold higher in GBM tissues compared to normal human brain tissue and correlated with glioma grade, while C18 ceramide was 5-fold lower in GBM tissues (Abuhusain et al., 2013). Fast-proliferating glioblastoma stem cells (GSCs) degrade ceramide and convert sphingosine to S1P more rapidly than slow-proliferating GSCs, and consequently release a significantly higher amount of S1P into the extracellular environment (Marfia et al., 2014). GBM cells and BTICs also release S1P which interacts with S1PRs influencing diverse cellular processes including cell migration, differentiation, proliferation, survival against apoptosis and angiogenesis (Zaibaq et al., 2022; Hawkins et al., 2020; Bassi et al., 2021). Moreover, overexpression of S1PR1-3 is observed in glioma cells (Hawkins et al., 2020). S1P also modulates EGFR expression in both cancer and non-cancer cells (Bassi et al., 2021).

Ceramide is considered a tumor suppressor lipid that can induce anti-proliferative and apoptotic responses in various tumor types, including gliomas (Zaibaq et al., 2022; Bassi et al., 2023). Ceramide levels are often reduced in gliomas due to increased degradation or altered synthesis and are significantly lower in HGG, correlating with malignant progression and poor patient survival (Riboni et al., 2002). The expression of various ceramide types and the balance among them are considered critical for different cancer cell progressions, including glioma. Generally, long-chained ceramides (C16, C18, C20) are considered as pro-apoptotic, while very-long chained ceramides (particularly C24 and C24:1) are associated with proliferative effects (Melero-Fernandez de Mera et al., 2022). In GBM tissue, C18-ceramide levels have been reported to decrease by up to 70%, while reconstitution of C18-ceramide induced cell death in human GBM cells resistant to TMZ (Das et al., 2018).

Recent findings indicate that IDH-wildtype gliomas exhibit a stronger imbalance in sphingolipid rheostat than IDH-mutant gliomas, resulting in elevated ceramide and sphingosine production, which may be the reason why IDH-mutant gliomas are less aggressive than IDH-wildtype (Dowdy et al., 2020). Moreover, several researches have shown that in IDH-mutant gliomas, the sphingolipid balance can be additionally shifted towards increasing pro-apoptotic ceramide production or towards decreasing pro-survival S1P production by targeting enzymes involved in the sphingolipid metabolism like SPHK1/2 and ceramidase, which reduces cancer hallmarks in IDH-mutant gliomas (Zaibaq et al., 2022; Dowdy et al., 2020; Tea et al., 2020) (Figure 2B).

### 4.1 Current improvements in sphingolipid rheostat targeting in glioma therapy

The sphingolipid rheostat can be altered by targeting enzymes that directly affect the ratio of ceramide to S1P, or by targeting other pathway enzymes that indirectly lead to increased ceramide or decreased S1P levels (Zaibaq et al., 2022; Tea et al., 2020). In gliomas, sphingolipid rheostat is out of balance promoting cell survival, thus providing a rich source of potential therapy targets. Ionizing radiation and TMZ, as current standard therapy for glioma patients, affect the sphingolipid pathway by activating SMPD1 which hydrolyzes sphingomyelin to pro-apoptotic ceramide (Hawkins et al., 2020; Tea et al., 2020).

Preclinical studies have shown that SPHK1 inhibitors can suppress glioma growth by reducing S1P levels. Still, their use in clinical trials is limited due to the conflicting data on the cytotoxic effects on different tumor cell models (Zaibaq et al., 2022; Sousa et al., 2023). Inhibition of SPHK1 selectively blocked angiogenesis, but it did not induce cell death while targeting SPHK1/2 both with dual SPHK inhibitor **SKI-II** proved to be more efficient in decreasing S1P production and promoting anticancer activity (Sousa et al., 2023; French et al., 2006). Additionally, SKI-II acts as a noncompetitive inhibitor of dihydroceramide desaturase 1 (DEGS1), an enzyme that catalyzes the introduction of a double bond into dihydroceramide, converting it into ceramide. The treatment of GBM cells with SKI-II resulted in the accumulation of dihydroceramide and depletion of S1P, which reduced cell proliferation and induced autophagy (Cingolani et al., 2014). SKI-II was found to have a synergistic effect with TMZ and induced cell death in the TMZ-resistant GBM cell lines (Sousa et al., 2023; Cingolani et al., 2014). Both SKI-II alone and combined with TMZ therapy are still in the preclinical testing phase (Sousa et al., 2023).

**ABC294640** (Opaganib) as the first-in-class selective competitive inhibitor of SPHK2 has no off-targets on protein kinases and decreases levels of S1P in tissue cultures (Lewis et al., 2018). Dowdy et al. (Dowdy et al., 2020) demonstrated that in IDH-mutant glioma subtypes, the combination of SPHK1 inhibitor ***N*,*N*-dimethylsphingosine** with **C17 sphingosine** reduced S1P production and enhanced ceramide and sphingosine accumulation. This induced growth arrest and apoptosis, specifically in IDH-mutant gliomas highlighting a metabolic vulnerability characterized by elevated ceramides and decreased SPHK2 expression in IDH-mutant compared to IDH-wildtype gliomas.

**Fenretinide**, a synthetic retinoid derivative, induces apoptosis in HGG cell lines by indirectly inhibiting DEGS1 (Puduvalli et al., 1999). Although additional testing led to a phase II clinical trial in patients with recurrent glioma and GBM, fenretinide was ineffective at the administered concentrations (Puduvalli et al., 1999; Puduvalli et al., 2004).

Preclinical studies and one pilot study have shown that **THC** (∆^9^-tetrahydrocannabinol) has biostatic effects on recurrent GBM by inhibiting DEGS1 while sparing non-transformed astroglial cells (Velasco et al., 2007; Del Pulgar et al., 2002). The combination of THC and CBD (cannabidiol) in 1:1 ratio (Sativex oral spray) with TMZ is currently in a phase II clinical trial (ARISTOCRAT trial) for GBM therapy (Bhaskaran et al., 2024).

Acid ceramidase (ASAH1) is the key enzyme that reduces ceramide levels by catalyzing its hydrolysis to sphingosine, thus also controlling the pool of sphingosine that can be converted to S1P (Doan et al., 2017a). It has been reported that ASAH1 is upregulated in GBM tissue and correlates with worse patient outcomes and radioresistance (Doan et al., 2017b). *In vitro* inhibition of ASAH1 enzyme with **carmofur**, a fluorouracil derivative, decreased the growth of TMZ-resistant GBM cells and effectively killed GSCs increasing ceramide levels and inducing apoptosis (Hawkins et al., 2023; Dementiev et al., 2019).

The most recent research by Navone et al (Navone et al., 2023) showed that **luteolin**, a natural flavonoid, inhibited the expression of SPHK1/2 while increasing the expression of both SGPL1 and CERS6, which decreased cell viability and survival of TMZ-resistant GSCs.

**Myriocin**, a competitive inhibitor of serine palmitoyltransferase 1 (SPTLC1), the first enzyme in *de novo* sphingolipid biosynthesis, can inhibit the proliferation of different cancer types by altering the sphingolipid rheostat (Lee et al., 2012; Yaguchi et al., 2017). Treating GBM cells with myriocin led to a 35% decrease in S1P levels (Bernhart et al., 2015). Synthetic sphingolipid analog, **fingolimod** (FTY720) was designed to improve the biostatic properties of myriocin, while also acting as a competitive inhibitor of SPHK2 (Zaibaq et al., 2022; Paugh et al., 2003). Fingolimod-1-phosphate inhibits HDAC1/2 and enhances histone acetylation leading to epigenetic regulation of specific genes that increase sensitivity to chemotherapeutics while also acting as an S1PR1 antagonist in the CNS lymph nodes (Hawkins et al., 2020; Pyne and Pyne, 2020). Carmofur, luteolin, myriocin, and fingolimod are still in the preclinical phase of research (Zaibaq et al., 2022).

Sphingomyelin is a crucial component of membrane lipid rafts essential for signaling. It is synthesized by sphingomyelin synthase 1 and 2 (SGMS1/2) isoenzymes, with SGMS1 being the primary isoform present at significantly lower levels in cancer cell membranes, including GBM, and reduced expression of SGMS1 in gliomas is associated with poorer prognostic outcomes (Fernández-García et al., 2019; Lladó et al., 2014).

Major progress in the therapy of glioma was achieved with synthetic **2-hydroxyoleic acid** (2-OHOA), which proved to be a highly specific activator of the SGMS1 isoform with a significantly higher bioavailability and lower toxicity compared to TMZ when administered in therapeutic doses (Lopez et al., 2023). It has been tested on glioma cell lines, preclinical animal studies, and clinical trials on patients are in phase I-II (Zaibaq et al., 2022; Fernández-García et al., 2019; Lopez et al., 2023). Currently, phase IIB-III trials are testing the combination of 2-OHOA, TMZ, and radiotherapy for GBM (NIHR, 2021), representing the closest a novel sphingolipid modulator has come to potential approval as a glioma therapy.

Recent preclinical research indicates that antidepressant **fluoxetine** (Prozac), a selective serotonin reuptake inhibitor (SSRI), can inhibit SMPD1 and induce cell death in glioma by accumulating sphingomyelin, which results in inhibition of oncogenic EGFR signaling and activating lysosomal stress (Bi et al., 2021). Combining fluoxetine, but not other SSRI inhibitors, with TMZ caused massive GBM cell death and complete tumor regression in mice, and it is now being tested in early phase I clinical trial (UCSD, 2024).

### 4.2 Future perspectives

Even though many exciting findings have been revealed in recent years regarding sphingolipid rheostat targeting in gliomas, they have still not been translated into viable therapy (Zaibaq et al., 2022). Due to the very complex and interconnected sphingolipid metabolism, it is very challenging to influence the activity of one enzyme or metabolic product and not disrupt the expression or metabolic fate of another. Further obstacles to the clinical application of sphingolipid-targeted therapies include challenges with drug delivery across the blood-brain barrier, lack of target specificity that leads to systemic toxicity, tumor heterogeneity (especially in GBMs), and the absence of reliable biomarkers to identify patients who would benefit most from sphingolipid-based treatments (Rahman and Ali, 2024). To overcome these challenges, research is focused on developing targeted delivery systems, combination therapies, and reliable biomarkers for patient selection (Janneh, 2024). While sphingolipid-targeted therapies represent a promising direction for glioma treatment, their potential can be further enhanced by integrating with existing therapeutic strategies. Combining SPHK inhibitors or ceramide analogs with standard treatments such as TMZ or immunotherapy may produce synergistic effects. Furthermore, the integration of genetic profiling in treatment by identifying specific sphingolipid enzyme profiles in individual tumors could enable a more personalized therapeutic approach (Scorsetti et al., 2022; Zhou et al., 2024). This personalized approach could be particularly valuable in IDH-mutant gliomas, where genetic variations in sphingolipid metabolism may influence treatment outcomes (Kayabolen et al., 2021). A still insufficiently studied field in sphingolipid-based glioma therapy is the role of ceramide variability, as different ceramide chain lengths may have opposing effects, despite the general association of ceramides with pro-apoptotic functions (Melero-Fernandez de Mera et al., 2022; Hartmann et al., 2012). Another interesting potential lies in the less abundant sphingoid base sphinganine (d18:0), although structurally similar to sphingosine, has distinct metabolic origins and lacks sphingosine’s apoptotic signaling abilities (Farley et al., 2024). On the other hand, sphinganine-1-phosphate exhibits strong signaling properties and binds to the S1PR1 even stronger than S1P, yet lacks S1P’s cytoprotective and proliferative effects (Van Brocklyn et al., 1998). The signaling roles of sphinganine and sphinganine-1-phosphate are yet to be clarified offering an interesting and unexplored field.

## 5 Conclusion

The sphingolipid rheostat is a critical regulator of cell fate in health and disease. In glioma cells, aberrant expression of enzymes of the sphingolipid metabolism leads the cells to escape apoptosis and promote survival and invasiveness. In recent years, several targeted therapies have been proposed that shift the rheostat towards apoptosis and tumor suppression. Targeting the sphingolipid rheostat for glioma treatment provides not only the first-line therapy options for gliomas but is also responsible for sensitizing particular types of gliomas, such as IDH-mutant glioma, for stronger chemotherapeutic response (Dowdy et al., 2020). Given the high complexity of gliomas, particularly GBM, and the limitations of treatment options available so far, a combination of targeting sphingolipid rheostat with some of the current therapies and with genetic profiling for personalized patient selection offers a promising approach to improve outcomes of patients suffering from these vicious tumors.
